# Self-care and risk reduction habits in older injection drug users with chronic wounds: a cross-sectional study

**DOI:** 10.1186/1477-7517-11-28

**Published:** 2014-10-19

**Authors:** Maria Elisa Smith, Natanya Robinowitz, Patrick Chaulk, Kristine E Johnson

**Affiliations:** Division of Infectious Diseases, Department of Medicine, Johns Hopkins Medical Institutions, 5200 Eastern Avenue, MFL Building, Center Tower, 3rd Floor, Baltimore, MD 21224 USA; Baltimore City Health Department, 1001 E. Fayette Street, Baltimore, MD 21202 USA

**Keywords:** Chronic wounds, Injection drug use, Aging, Harm reduction

## Abstract

**Background:**

We surveyed a population of injection drug users (IDUs) frequenting the mobile Baltimore City Needle Exchange Program (BNEP) to investigate self-care factors associated with chronic wounds, a significant cause of morbidity especially among older IDUs.

**Methods:**

Participants ≥18 years old completed a survey regarding chronic wounds (duration ≥8 weeks), injection and hygiene practices. Study staff visually verified the presence of wounds. Participants were categorized into four groups by age and wound status. Factors associated with the presence of chronic wounds in participants ≥45 years were analyzed using logistic regression.

**Results:**

Of the 152 participants, 19.7% had a chronic wound. Of those with chronic wounds, 18 were ≥45 years old (60.0%). Individuals ≥45 years old with chronic wounds were more likely to be enrolled in a drug treatment program (Odds ratio (OR) 3.4, 95% Confidence interval (CI) 1.0–10.8) and less likely to use cigarette filters when drawing up prepared drug (OR 0.2, 95% CI 0.03–0.7) compared to the same age group without chronic wounds. Compared to individuals <45 years old without chronic wounds, individuals ≥45 with a chronic wound were more likely to report cleaning reused needles with bleach (OR 10.7, 95% CI 1.2–93.9) and to use the clinic, rather than an emergency room, as a primary source of medical care (OR 3.4, 95% CI 1.1–10.4).

**Conclusions:**

Older IDUs with chronic wounds have different, and perhaps less risky, injection and hygiene behaviors than their peers and younger IDUs without wounds in Baltimore City. Because of these differences, older IDUs with wounds may be more receptive to community-based healthcare and substance abuse treatment messages.

## Background

Older injection drug users (IDUs) are a growing subgroup of active IDUs and IDUs seeking treatment [1–5]. It is estimated that the number of adults 50 years or older with substance use problems will double from an annual average of 2.8 million (2002–06) to 5.7 million by 2020 [6]. Age-associated health complications and ongoing chronic drug use may further intensify the social marginalization of older IDUs, which may diminish quality of life, access to health care and social productivity [7, 8]. Furthermore, injection drug use has been associated with accelerated biologic aging [9, 10] and the premature onset of health conditions normally associated with aging [3, 11, 12]. Due to these life course differences and evidence that IDUs have higher overall mortality rates, older IDUs are of growing interest to public health researchers and are typically defined as individuals older than 40–50 years [13, 14].

Overall, older IDUs report impaired mental and physical health and functioning compared to age- and gender-matched population standards [15, 16]. Older IDUs often self-reported health conditions such as deep vein thrombosis, skin ulcers, respiratory problems, diabetes, hypertension, hepatitis, and liver cirrhosis [8, 15]. Studies on venous damage and chronic venous insufficiency (CVI) in IDUs revealed that CVI develops prematurely in IDUs [11, 12].

Certain risky injection drug use behaviors such as sharing needles, subcutaneous injection (skin-popping), injecting a mixture of heroin and cocaine (speedballing), using cigarette filters to draw up prepared drug, and injection under unhygienic conditions, have been linked to adverse outcomes such as abscesses, skin and soft tissue infections, injection site ulcers, and endocarditis [17–25]. Local and national public health authorities have implemented harm reduction measures to raise awareness and to educate IDUs regarding these risky behaviors. However, efforts have not been focused on raising awareness of the long-term consequences of injection-related venous damage among IDUs, which can lead to CVI and lower extremity ulcers. The effectiveness of these programs may also be attenuated by an individual’s severity of addiction, socio-cultural environment and prior exposure to harm reduction messages [5].

Some studies have determined that older IDUs who started injecting at a young age, as opposed to later in adulthood, were more likely to have high-risk injection practices including sharing of injection equipment and more frequent injection [26, 27]. In addition, the existing literature regarding the aging IDU population focuses on general physical and mental health conditions or injection-related behaviors [13, 15, 16, 26–29]. The injection behaviors and socio-demographic characteristics of IDUs living with chronic wounds have not been well explored, even though chronic wounds are a significant health concern among older active or former IDUs.

We examined the prevalence of chronic wounds and associated injection-related behaviors among an IDU population accessing needle exchange services from the Baltimore City mobile Needle Exchange Program (BNEP), which has been in operation since 1994 [30]. We were interested specifically in determining whether there were distinctions between the demographics and behaviors of older IDUs living with chronic wounds as compared to their peers and younger IDUs. An improved understanding of the behaviors of older IDUs may help inform prevention strategies and both skin- and drug-related treatment efforts for individuals living with chronic wounds.

## Methods

We conducted a cross-sectional study among active IDU participants of the BNEP, age ≥18 years, regardless of wound status. Data collection occurred between May 2012 and November 2013 and was conducted at five different exchange sites, though most participants were from Site A and Site B, which were 1.9 miles apart and demographically distinct. Site A is frequented primarily by African Americans and Site B is frequented primarily by Caucasians. Site C was near several local exotic dance clubs and was geographically separate from Sites A and B.

All participants provided written, informed consent and completed a paper-administered survey including questions addressing demographics, injection behaviors, pre-injection skin care, wound history, wound care, and general medical history. Surveys required approximately 20 minutes and upon completion, participants were given a $10 gift card for local businesses. Chronic wounds were defined as open areas on the skin that had been present and non-healing for ≥8 weeks. Study team members visually verified the presence of chronic wounds at the time of the survey. This study was approved by the Johns Hopkins Medicine Institutional Review Board.

Participants were stratified into four groups by reported age (<45 years or ≥45 years) and wound status (presenting with a current chronic wound or without a current chronic wound). The primary outcome was considered the presence of a chronic wound among participants ≥45 years. This outcome was independently compared to three distinct reference groups: 1) participants < 45 years *without* chronic wounds; 2) participants ≥45 years *without* chronic wounds; 3) participants < 45 years *with* chronic wounds.

All variables were categorical except for age and number of times a needle was used, which were continuous variables. Predictor variables associated with the primary outcome group were analyzed using univariate and multivariate logistic regression. Odds ratios predicting risk of outcomes of interest with 95% confidence intervals were assessed. T-tests were used to compare means and/or proportions. *P* values ≤ 0.05 were considered statistically significant. Statistical analysis was performed using Stata 12 (StataCorp, College Station, TX).

## Results

A total of 152 individuals were surveyed. The overall prevalence of chronic wounds was 19.7% (n/N = 30/152). Of the 152 individuals, 73 were younger than 45 years (48.0%) and 79 were aged 45 years or older (52.0%). Of those with chronic wounds, 12 were in individuals younger than 45 years (40.0%) and 18 were in individual aged 45 years or older (60.0%) (*p* = 0.121). We focused our analysis on the latter subgroup of participants: IDUs who were 45 years of age or older with at least one current chronic wound. This group accounted for 11.8% of all study participants (18/152).

Within the particular subgroup of interest (IDUs age ≥45 years with chronic wound(s)), the median age was 55 years with an interquartile range (IQR) of 46–58. The participants were primarily male (61.1%), African American (72.2%), reported having stable housing (94.4%), and had a self-reported HIV prevalence rate of 27.8% (5/18). They reported long histories with injection drug use, most having injected for greater than 15 years (94.4%). The most frequently reported injection behaviors included daily heroin injection (72.2%), daily speedball injection (55.6%), leg injection (61.1%) and cleaning the injection site with alcohol before injecting (72.2%). Of the nine participants who reported reusing their needles (9/18, 50.0%), seven of eight reported cleaning their needles with bleach prior to reuse (87.5%). Older IDUs with chronic wounds also reused their needles less frequently compared to their peers without wounds (*p* = 0.021) and compared to younger IDUs without wounds (*p* < 0.001). See Table 1 for additional information on demographics, injection practices and medical history for all participants, participants in the outcome group (IDUs age ≥45 years with chronic wound(s)), and participants in the three reference groups.

**Table 1:** **Socio-demographic, medical and injection behaviors by age and chronic wound status †**

	All participants	Outcome group ≥45 years, with chronic wound	Reference group #1 <45 years, no chronic wound		Reference group #2 ≥45 years, no chronic wound		Reference group #3 < 45 years, with chronic wound	
Description	No. (%)	No. (%)	No. (%)	***p*** ^a^	No. (%)	***p*** ^a^	No. (%)	***p*** ^a^
	N = 152	N = 18	N = 61		N = 61		N = 12	
All participants (N = 152)	152 (100.0)	18 (11.8)	61 (40.1)	…	61 (40.1)	…	12 (7.9)	…
Age^b,c^	45; (35–52)	55; (46–58)	33; (28–39)	<0.001*	52; (49–57)	0.579	40; (35–42)	<0.001*
Gender								
Male	96 (63.2)	11 (61.1)	31 (50.8)	0.442	48 (78.7)	0.131	6 (50.0)	0.548
Female	56 (36.8)	7 (38.9)	30 (49.2)	0.442	13 (21.3)	0.131	6 (50.0)	0.548
Race								
Caucasian	75 (49.3)	4 (22.2)	47 (77.1)	<0.001*	16 (26.2)	0.701	8 (66.7)	0.015*
African American	68 (44.7)	13 (72.2)	9 (14.8)	<0.001*	44 (72.1)	0.926	2 (16.7)	0.003*
Native American	4 (2.6)	1 (5.6)	1 (1.6)	0.379	0 (0.0)	0.063	2 (16.7)	0.322
Other or Multiple^d^	5 (3.3)	0 (0.0)	4 (6.6)	0.263	1 (1.6)	0.589	0 (0.0)	…
Housing^e^								
Stable	113 (74.3)	17 (94.4)	41 (67.2)	0.022*	47 (77.1)	0.100	8 (66.7)	0.046*
Unstable	39 (25.7)	1 (5.6)	20 (32.8)	0.022*	14 (23.0)	0.100	4 (33.3)	0.046*
Exchange site								
Site A	54 (35.5)	9 (50.0)	10 (16.4)	0.003*	34 (55.7)	0.670	1 (8.3)	0.018*
Site B	72 (47.4)	5 (27.8)	40 (65.6)	0.004*	18 (29.5)	0.889	9 (75.0)	0.011*
Site C	5 (3.3)	0 (0.0)	4 (6.6)	0.263	0 (0.0)	–	1 (8.3)	0.214
Other	21 (13.8)	4 (22.2)	7 (11.5)	0.249	9 (14.8)	0.457	1 (8.3)	0.316
Jail/prison - past 6 months	45 (29.6)	1 (5.6)	28 (45.9)	0.002*	12 (19.7)	0.157	4 (33.3)	0.046*
Years injecting drugs								
≤2	15 (9.9)	0 (0.0)	12 (19.7)	0.041*	2 (3.3)	0.435	1 (8.3)	0.214
3 – 8	25 (16.4)	0 (0.0)	18 (29.5)	0.009*	5 (8.2)	0.209	2 (16.7)	0.073
9 – 14	20 (13.2)	1 (5.6)	12 (19.7)	0.157	5 (8.2)	0.715	2 (16.7)	0.322
≥15	92 (60.5)	17 (94.4)	19 (31.2)	<0.001*	49 (80.3)	0.157	7 (58.3)	0.016*
Injection site location								
Arm	92 (60.5)	7 (38.9)	38 (62.3)	0.078	39 (63.9)	0.059	8 (66.7)	0.136
Leg	37 (24.3)	11 (61.1)	8 (13.1)	<0.001*	16 (26.2)	0.006*	2 (16.7)	0.016*
Neck	17 (11.2)	4 (22.2)	10 (16.4)	0.571	2 (3.3)	0.008*	1 (8.3)	0.317
Drug treatment program^f^	36 (23.7)	8 (53.3)	12 (20.7)	0.012*	15 (25.4)	0.037*	1 (11.1)	0.039*
Using cigarette filter^g^	65 (42.8)	2 (11.8)	31 (51.7)	0.003*	28 (46.7)	0.009*	4 (40.0)	0.089
Using needles (# of times)^h^	3; 1 – 4	1; 1 – 3	3; 2 – 7	<0.001*	2; 1 – 3	0.021*	3; 2 – 3	0.112
Clean needles upon reuse^i^								
Water	56 (50.5)	1 (12.5)	29 (60.4)	0.012*	23 (51.1)	0.043*	3 (30.0)	0.375
Bleach	55 (49.5)	7 (87.5)	19 (39.6)	0.012*	22 (48.9)	0.043*	7 (70.0)	0.375
Cleaning injection site^j^								
Do not clean	35 (23.0)	4 (22.2)	13 (21.3)	0.935	12 (19.7)	0.817	6 (50.0)	0.114
Water	12 (7.9)	0 (0.0)	4 (6.6)	0.263	7 (11.5)	0.132	1 (8.3)	0.214
Soap/water	14 (9.2)	0 (0.0)	9 (14.8)	0.083	4 (6.6)	0.263	1 (8.3)	0.214
Alcohol	83 (54.6)	13 (72.2)	33 (54.1)	0.171	34 (55.7)	0.210	3 (25.0)	0.011*
Multiple or other	8 (5.3)	1 (5.6)	2 (3.3)	0.655	4 (6.6)	0.879	1 (8.3)	0.772
Medical care provider								
Clinic	48 (31.6)	9 (50.0)	14 (23.0)	0.027*	23 (37.7)	0.350	2 (16.7)	0.064
Private doctor	14 (9.2)	1 (5.6)	5 (8.2)	0.715	6 (9.8)	0.581	2 (16.7)	0.322
Emergency room	85 (55.9)	8 (44.4)	42 (68.9)	0.058	28 (45.9)	0.911	7 (58.3)	0.456
Other	5 (3.3)	0 (0.0)	0 (0.0)	–	4 (6.6)	0.263	1 (8.3)	0.214

† All numbers have been rounded to nearest decimal place.

^a^ *p* values refer to the comparison between the Reference group and the Outcome Group. *p* value for "Age" was obtained using two-group mean comparison t-test. All other *p* values were obtained using two-group proportion t-test.

^b^Continuous variable.

^c^Median; (Interquartile range).

^d^Hispanic, Asian, multiple.

^e^Unstable includes living in a shelter, on the streets, in an abandoned unit, no set place, or multiple. Stable housing includes living in an owned/rented house, subsidized housing, with a friend, other (e.g. transitional house).

^f^Participant in a drug treatment program; Denominator changes where responses were missing: All: N = 152; Outcome group: N = 15; Reference group #1: N = 58; Reference group #2: 59; Reference group #3: N = 9.

^g^Using cigarette filters to draw up prepared drug; Denominator changes where responses were missing: All: N = 152; Outcome group: N = 17; Reference group #1: N = 60; Reference group #2: 60; Reference group #3: N = 10.

^h^Denominator changes where responses were missing: All: N = 150; Outcome group: N = 17; Reference group #1: N = 61; Reference group #2: 60; Reference group #3: N = 12.

^i^Includes only participants who reported reusing needles. All: N = 111; Outcome group: N = 8; Reference group #1: N = 48; Reference group #2: 45; Reference group #3: N = 10.

^j^Most frequent method of cleaning injection site before injection; "Other" includes bleach, saliva, baby wipe, multiple agents.

*p ≤ 0.05.

### Older IDUs with chronic wounds compared to younger IDUs without wounds

Compared to individuals younger than 45 years who did not have chronic wounds, the univariate analysis indicated that participants older than 45 years old with chronic wounds were more likely to be African American compared to Caucasian (Odds ratio (OR) 17.0, 95% Confidence interval (CI) 4.5 – 64.1, *p* < 0.001), to have stable housing (OR 8.3, 95% CI 1.0–66.8, *p* = 0.047), to visit a clinic as their primary source of medical care rather than an emergency room (OR 3.4, 95% CI 1.1–10.4, *p* = 0.035), and to frequent BNEP site A versus Site B (OR 7.2, 2.0–26.3, *p* = 0.003) (Table 2A). They were also more likely to have injected drugs for 15 or more years (OR 10.7, 95% CI 1.3–91.5, *p* = 0.030), to be participating in a drug treatment program (OR 4.4, 95% CI 1.3–14.5, *p* = 0.016), to inject speedball everyday (OR 4.2, 95% CI 1.1–15.7, *p* = 0.035), to inject into the leg (OR 10.4, 95% CI 3.1–34.7, *p* = <0.001) and to clean needles with bleach upon reuse (OR 10.7, 95% CI 1.2–93.9, *p* = 0.033). Additionally, they were less likely to have been in jail or prison for more than 24 hours during the past 6 months (OR 0.07, 95% CI 0.009–0.6, *p* = 0.012), and also less likely to use cigarette filters to draw up prepared drug (OR 0.1, 95% CI 0.03–0.6, *p* = 0.009).

**Table 2:** **Group #1: Older IDUs with chronic wounds compared to younger IDUs without wounds**

	A. univariate (N = 79)			B. multivariate ^a^(N = 71)		
	OR	95% CI	***p***	OR	95% CI	***p***
Race						
Caucasian	Ref			Ref		
African American	17.0	4.5 – 64.1	<0.001*	16.3	1.4 – 190.3	0.026*
Native American	11.8	0.6 – 225.4	0.102	9.3	0.2 – 475.3	0.268
Other	…^b^					
Housing^c^						
Unstable	Ref					
Stable	8.3	1.0 – 66.8	0.047*	…^d^		
Medical Care						
Emergency room	Ref					
Clinic	3.4	1.1 – 10.4	0.035*	…^d^		
Private doctor	1.1	0.1 – 10.2	0.996			
Other	…^b^					
Exchange site (N = 29)						
Site A	Ref			Ref		
Site B	7.2	2.0 – 26.3	0.003*	1.5	0.2 – 12.0	0.719
Site C	4.6	1.0 – 21.3	0.053*	17.8	1.2 – 254.5	0.034*
Time injecting drugs (years)						
9 – 14	Ref					
≥15	10.7	1.3 – 91.5	0.030*	…^d^		
Participating in drug treatment program						
No	Ref					
Yes	4.4	1.3 – 14.5	0.016*	…^d^		
Speedball						
Never	Ref					
Occasionally	1.2	0.3 – 5.3	0.820	…^d^		
Everyday	4.2	1.1 – 15.7	0.035*			
Injecting into leg						
No	Ref			Ref		
Yes	10.4	3.1 – 34.7	<0.001*	9.9	1.3 – 73.1	0.024*
Skin-popping						
No	Ref					
Yes	4.4	1.0 – 19.9	0.055	…^d^		
Cleaning needle with bleach upon reuse						
Water	Ref					
Bleach	10.7	1.2 – 93.9	0.033*	…^d^		
Jail/Prison during last 6 months						
No	Ref					
Yes	0.07	0.009 – 0.6	0.012*	…^d^		
Cigarette filters to draw up drug						
No	Ref			Ref		
Yes	0.1	0.03 – 0.6	0.009*	0.06	0.004 – 0.9	0.039*

Abbreviations: *IDUs* injection drug users, *OR* odds ratio, *CI* confidence interval.

^a^Variables with at least one category with *p* ≤ 0.010 at the univariate level were included in the multivariate model.

^b^Insufficient observations for both univariate and multivariate logistic regression.

^c^Unstable includes living in a shelter, on the streets, in an abandoned unit, no set place, or multiple. Stable housing includes living in an owned/rented house, subsidized housing, with a friend, other (e.g. transitional house).

^d^Not included in multivariate model.

*p ≤ 0.05.

In the multivariate model, older individuals with wounds were more likely to be African American (Adjusted odds ratio (AOR) 16.3, 95% CI 1.4–190.3, *p* = 0.026) and inject into the leg (AOR 9.9, 95% CI 1.3–73.1, *p* = 0.024), and they were less likely to use cigarette filters (AOR 0.06, 95% CI 0.004–0.9, *p* = 0.039) (Table 2B).

### Older IDUs with chronic wounds compared to similar-aged peers without wounds

Among participants 45 years or older, those with a chronic wound were more likely to be in a drug treatment program (OR 3.4, 95% CI 1.0–10.8, *p* = 0.043), and to inject into the leg (OR 4.4, 95% CI 1.5–13.4, *p* = 0.008) or neck (OR 8.4, 95% CI 1.4–50.7, *p* = 0.020), compared to the same age group without chronic wounds (Table 3A). In the multivariable model, older individuals with chronic wounds remained less likely to use cigarette filters when drawing up prepared drug into the syringe (AOR 0.1, 95% CI 0.01–0.9, *p* = 0.044) (Table 3B).

**Table 3:** **Group #2: Older IDUs with chronic wounds compared to older IDUs without wounds**

	A. univariate (N = 79)			B. multivariate ^a^(N = 72)		
	OR	95% CI	***p***	OR	95% CI	***p***
Trading sex for money						
No	Ref			Ref		
Yes	3.3	1.1 – 10.0	0.039*	2.3	0.4 – 13.9	0.346
Participating in drug treatment program						
No	Ref			Ref		
Yes	3.4	1.0 – 10.8	0.043*	3.2	0.8 – 12.9	0.098
Injecting into leg						
No	Ref			Ref		
Yes	4.4	1.5 – 13.4	0.008*	3.2	0.7 – 13.4	0.120
Injecting into neck						
No	Ref			Ref		
Yes	8.4	1.4 – 50.7	0.020*	4.9	0.4 – 58.3	0.212
Using cigarette filters to draw up drug						
No	Ref			Ref		
Yes	0.2	0.03 – 0.7	0.018*	0.1	0.01 – 0.9	0.044*

Abbreviations: *IDUs* injection drug users, *OR* odds ratio, *CI* confidence interval.

^a^Variables with at least one category with *p* ≤ 0.050 at the univariate level were included in the multivariate model.

*p ≤ 0.05.

### Older IDUs with chronic wounds compared to younger IDUs with wounds

Among participants with chronic wounds, those 45 years or older were more likely to be African American (OR 13.0, 95% CI 1.9–88.0, *p* = 0.009), to exchange needles at Site A compared to Site B (OR 16.2, 95% CI 1.6–167.7, *p* = 0.020), to inject into the leg (OR 7.9, 95% CI 1.3–47.0, *p* = 0.024), and to clean the injection site with alcohol before injection (OR 6.5, 95% CI 1.1–38.6, *p* = 0.040) when compared to younger IDUs with chronic wounds (Table 4A). In the multivariate model, leg injection was more common among older individuals with chronic wounds (AOR 31.3, 95% CI 1.1–873.6, *p* = 0.043) (Table 4B).

**Table 4:** **Group #3: Older IDUs with chronic wounds compared to younger IDUs with wounds**

	A. univariate (N = 30)			B. multivariate ^a^(N = 21)		
	OR	95% CI	***p***	OR	95% CI	***p***
Race						
Caucasian	Ref			Ref		
African American	13.0	1.9 – 88.0	0.009*	4.3	0.06 – 290.1	0.502
Native American	1.0	0.07 – 14.6	1.000	0.1	0.001 – 12.7	0.387
Other	…^b^					
Exchange site (N = 29)						
Site A	Ref			Ref		
Site B	16.2	1.6 – 167.7	0.020*	2.0	0.03 – 146.2	0.751
Site C	7.2	0.6 – 83.3	0.114	…^b^		
Injecting into leg						
No	Ref			Ref		
Yes	7.9	1.3 – 47.0	0.024*	31.3	1.1 – 873.6	0.043*
Cleaning injection site before injection (N = 26) ^c^						
Do not clean	Ref			Ref		
Alcohol	6.5	1.1 – 38.6	0.040*	13.5	0.5 – 390.6	0.130

*Abbreviations*: OR odds ratio, CI confidence interval.

^a^Variables with at least one category with *p* ≤ 0.050 at the univariate level were included in the multivariate model.

^b^Insufficient observations for logistic regression

^c^Other categories within this variable were dropped in univariate and multivariate analysis due to insufficient observations. Dropped variables included water, soap/water, multiple or other.

*p ≤ 0.05.

## Discussion

The high prevalence of chronic wounds in our community-recruited IDU study population (19.7%) and the disproportionate overrepresentation of older IDUs with chronic wounds (60%) suggests the need for focused interventions to improve awareness of injection-related venous damage and chronic wounds in IDUs to better prevent and treat this condition.

Upon comparing older IDUs with chronic wounds to their same-aged peers without chronic wounds and to younger IDUs with and without wounds, this group of older individuals with wounds appeared to engage in behaviors that were more consistent with self-care habits and less-risky injection practices. For example, they were more likely to have stable housing, use a clinic rather than an emergency room as the primary source of medical care, participate in a drug treatment program, clean needles with bleach, and clean their injection site with alcohol before injection. They were also less likely to have been recently in jail or prison, or to have used cigarette filters to draw up prepared drug. Use of cigarette filters, rather than needle exchange-distributed packed cotton filters, is considered riskier behavior and generally discouraged by harm reduction advocates because it is less effective at filtering particles out of the drug and can be associated with endocarditis or phlebitis [23, 31]. It is possible that older IDUs with wounds are in a different stage of life, as they have survived for many years with substance abuse issues and have perhaps learned to cope with their substance abuse and chronic health conditions better than younger IDUs. It remains to be determined if these differences in behaviors surrounding injection and cleanliness are a result of living with a chronic wound, maturing as an injection drug user and/or the internalization of the syringe exchange program’s ongoing harm reduction messages and education.

Older IDUs with wounds were more likely to be African American even after adjusting for exchange site. In contrast, younger IDUs were more likely to be Caucasian. These findings may be a reflection of the drug use patterns in Baltimore City and at the BNEP sites that were surveyed. This suggests that there are racial and/or socio-cultural differences among younger Caucasian IDUs that may lend preference for certain, and perhaps riskier, injection-related and other destabilizing behaviors that may lead to increased rates of homelessness and incarceration, among other factors, that we observed in this analysis. Understanding these differences within local subgroups of an IDU population may facilitate more targeted harm reduction efforts.

Other findings from this study are consistent with a population engaged in long-term injection drug use, which can lead to venous scarring and venous disease. As IDUs mature in their injection drug use habits, there may be increasing reliance on injecting into veins in other locations such as the leg or neck, as we observed among the older IDUs with wounds. Chronic wounds appear to be associated with prolonged drug use, even among the younger IDUs, as the majority of younger IDUs with chronic wounds had also been injecting for at least 15 years. Injecting into the leg together with prolonged injection drug use, resultant venous damage and physiologic aging of the veins, likely puts older IDUs at heightened risk for both CVI and lower extremity ulcers [32].

Our study had limitations. Participants were active members of the BNEP, and therefore they may report different injection and skin care practices than IDUs not accessing community-based harm reduction services and education. The survey also relied upon self-reported data. Additionally, there were limitations associated with the small sample size of individuals with chronic wounds, however the overall cohort size and survey design was sufficient to characterize some behaviors as truly distinct in the population with chronic wounds. Despite these potential limitations, we report for the first time self-care behaviors among older IDUs living with chronic wounds in a mobile metropolitan needle exchange program in Baltimore City.

## Conclusions

Our findings suggest that older IDUs with chronic wounds have different, and perhaps less risky, injection and hygiene practices than their peers and younger IDUs without wounds. Moreover, older IDUs with chronic wounds may represent a different demographic among those using illicit substances and a distinct IDU subgroup that may be more receptive to local healthcare and/or substance abuse treatment messages. This subgroup may also be more likely to engage in meaningful relationships and therapeutic alliances with medical providers to address drug use and/or health conditions previously neglected.

Future public health and harm reduction interventions to raise awareness of the known associations between injection practices, venous damage, and chronic wounds among IDUs who have recently initiated injecting and among IDUs who have a prolonged injection history may represent valuable measures to prevent long-term wound-associated morbidity and disability. Harm reduction programs should consider including such educational information and referrals to local wound care centers in their regular encounters with IDUs.

## Authors’ information

MES has a Bachelor of Science in Molecular and Cellular Biology and a Bachelor of Arts in History of Science, Technology and Medicine from the Johns Hopkins University. She is currently a research assistant for KEJ at the Johns Hopkins Medical Institutions.

NR obtained her Master of Science in Public Health from the Johns Hopkins Bloomberg School of Public Health. She has extensive experience with harm reduction efforts and works for the Baltimore City Health Department’s Community Risk Reduction Services.

PC, MD, MPH, is the Acting Deputy Commissioner, Division of Disease Control, Baltimore City Department of Health and has extensive experience in congressional, state, and municipal health and public health public policy.

KEJ, MD, MSc is Assistant Professor of Medicine in the Division of Infectious Diseases at the Johns Hopkins University School of Medicine. She completed a residency in Internal Medicine at the University of Colorado and a fellowship in Infectious Diseases at Johns Hopkins University. She has significant experience in translational research and public health program implementation. She is the primary physician for the BNEP’s wound care clinic and for this research study.

## Abbreviations

IDUs: Injection drug users
CVI: Chronic venous insufficiency
IQR: Interquartile range
OR: Odds ratio
CI: Confidence interval
AOR: Adjusted odds ratio.
